# High-Molecular-Weight PLA-*b*-PEO-*b*-PLA Triblock Copolymer Templated Large Mesoporous Carbons for Supercapacitors and CO_2_ Capture

**DOI:** 10.3390/polym12051193

**Published:** 2020-05-23

**Authors:** Mohamed Gamal Mohamed, Wei-Shih Hung, Ahmed F. M. EL-Mahdy, Mahmoud M. M. Ahmed, Lizong Dai, Tao Chen, Shiao-Wei Kuo

**Affiliations:** 1 Department of Materials and Optoelectronic Science, Center of Crystal Research, National Sun Yat-Sen University, Kaohsiung 80424, Taiwan; mgamal.eldin12@yahoo.com (M.G.M.); m063100027@student.nsysu.edu.tw (W.-S.H.); ahmed1932005@gmail.com (A.F.M.E.-M.); kuosw19760501@gmail.com (M.M.M.A.); 2 Fujian Provincial Key Laboratory of Fire Retardant Materials, College of Materials, Xiamen University, Xiamen 361005, China; lzdai@xmu.edu.cn; 3 Ningbo Institute of Material Technology and Engineering, Chinese Academy of Science, Zhongguan West Road 1219, Ningbo 315201, China; tao.chen@nimte.ac.cn; 4 Department of Medicinal and Applied Chemistry, Kaohsiung Medical University, Kaohsiung 807, Taiwan

**Keywords:** hydrogen bonding, triblock copolymer, CO_2_ capture, supercapacitors and mesoporous carbon

## Abstract

High-molecular-weight PLA_440_-*b*-PEO_454_-*b*-PLA_440_ (LEL) triblock copolymer was synthesized through simple ring-opening polymerization (ROP) by using the commercial homopolymer HO-PEO_454_-OH as the macro-initiator. The material acted as a single template to prepare the large mesoporous carbons by using resol-type phenolic resin as a carbon source. Self-assembled structures of phenolic/LEL blends mediated by hydrogen bonding interaction were determined by FTIR and SAXS analyses. Through thermal curing and carbonization procedures, large mesoporous carbons (>50 nm) with a cylindrical structure and high surface area (>600 m^2^/g) were obtained because the OH units of phenolics prefer to interact with PEO block rather than PLA block, as determined by FTIR spectroscopy. Furthermore, higher CO_2_ capture and good energy storage performance were observed for this large mesoporous carbon, confirming that the proposed approach provides an easy method for the preparation of large mesoporous materials.

## 1. Introduction

The high surface areas and pore volumes of porous materials are very interesting for different applications, including energy storage, adsorption, drug delivery, and catalysis [1,2,3,4,5,6,7,8,9,10,11,12,13]. Based on their pore sizes, three different kinds of porous materials are defined from the IUPAC rules, including microporous (<2 nm), mesoporous (2–50 nm), and macroporous (>50 nm) materials.

In general, the preparation of mesoporous silicas, phenolic resin, or carbons templated by block copolymer is the most common approach compared with foaming, phase separation, hard-template, and molecular imprinting methods [14,15,16,17]. Using this approach, well-defined mesoporous structures can be obtained since the block copolymer could be self-assembled into different structures through microphase separation connected by covalent bond [18,19,20]. The concept of mediating the hydrogen bonding strength in block copolymer mixtures [21,22,23,24,25,26,27,28,29,30,31,32] to synthesize mesoporous materials has been widely discussed in our previous studies [18,19,33,34,35]. Using a commercial pluronic-type poly(ethylene oxide–*b*–propylene oxide–*b*–ethylene oxide) triblock copolymer as the template is the most widely used approach to preparing mesoporous materials; however, because of the limitation presented by the molecular weight of this kind of triblock copolymer, it is difficult to prepare mesoporous phenolic or carbon with pore size >10 nm [36,37,38,39,40,41]. As a result, using a high-molecular-weight long hydrophobic block in a PEO-based block copolymer including poly(ethylene-oxide-*b*-styrene) (PEO-*b*-PS) [42,43,44], poly(ethylene oxide-*b*-caprolactone) (PEO-*b*-PCL) [33,34,35,45,46,47], poly(ethylene-oxide-*b*-methyl methacrylate) (PEO-*b*-PMMA) [48], or poly(ethylene oxide–*b*–lactic acid) (PEO-*b*-PLA) [49,50] as the templates is the easiest way to prepare large mesoporous materials. For example, employing PEO_125_-*b*-PS_230_ and PEO_114_-*b*-PLA_94_ diblock copolymers as a template could provide the large pore size (23 and 21 nm, respectively) required to prepare mesoporous carbon [42,50]. In addition, the high molecular weight of PEO-*b*-PS-*b*-PI triblock copolymer (ca. 100 kDa) as a single template was used to obtain a mesoporous carbon with large pore size (39 nm) [51].

Zhao et al. proposed another method to further increase the pore size of mesoporous materials by using PEO-*b*-PMMA/PMMA or PEO-*b*-PS/PS blend as a co-template to obtain large mesoporous materials, in which either the homopolymer PMMA or PS could act as the pore expander. However, macrophase separation may occur at higher homopolymer concentrations (>20 wt.%) that would induce disordered mesoporous materials with multimodal pore sizes (ca. 40–90 nm) [52,53]. Furthermore, the high-molecular-weight PEO-*b*-PMMA and PEO-*b*-PS diblock copolymers are difficult to synthesize from the chain end modification of the PEO block segment (such as PEO-Br) by atom transfer radical polymerization [42,48]. Therefore, the high-molecular-weight triblock copolymer PCL-*b*-PEO-*b*-PCL (ca. 120 kDa) was synthesized through simple ring-opening polymerization (ROP) using simple HO-PEO_454_-OH as the macro-initiator in our previous study [54]. A large number of macroporous carbons with a pore size larger than 50 nm could be obtained using this PCL-*b*-PEO-*b*-PCL triblock copolymer, in which the resol phenolic resin also acts as the carbon source [54]. We also observed that the hydrogen bonding interaction between phenolic resin and those hydrophobic block segments played an important role in the determination of the pore size of mesoporous carbons [50]. For example, the weak hydrogen bonding interaction of PCL (inter-association equilibrium constant (*K*_A_) = 116) [55] and PMMA (*K*_A_ = 20) segment [56,57] induced thicker walls but smaller pore size in mesoporous carbons as compared with PS or PLA segments having similar molecular weight. This phenomenon was due to the lack of hydrogen bonding interaction between the phenolic and PS segment. Thus, the PS or PLA segment could undergo complete microphase separation and induce thinner walls, but a larger pore size of mesoporous carbons could only be obtained after the template was removed [50].

**Scheme 1:**
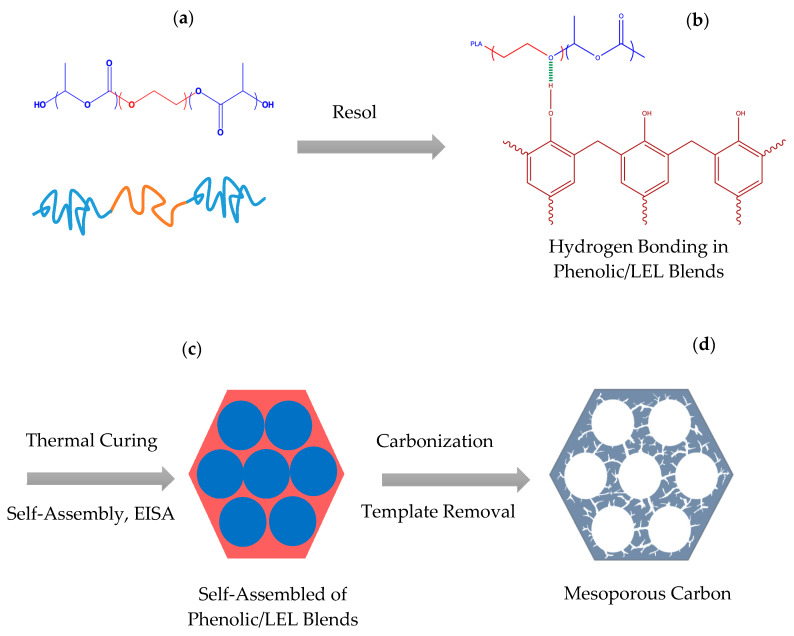
(**a**) Chemical structure of PLA_440_-*b*-PEO_454_-*b*-PLA_440_ (LEL) triblock copolymer; (**b**) hydrogen bonding interaction in phenolic/LEL blends; (**c**) thermal-curing-induced self-assembled structure of phenolic/LEL blends; (**d**) fabrication of mesoporous carbon after carbonization.

In this study, we also synthesized a relatively higher molecular weight PLA440-b-PEO454-b-PLA440 (LEL) triblock copolymer using HO-PEO454-OH as the macro-initiator by simple ROP. Compared with the PEO114-b-PLA94 diblock copolymer [50], this LEL triblock copolymer possessed high molecular weight as a template and thus could provide large mesoporous carbons as expected. In addition, as also compared with PCL-b-PEO-b-PCL triblock copolymer, using the PLA-b-PEO-b-PLA triblock copolymer as the template had two advantages, including: (1) No crystallization of the PLA segment of the template occurred since the self-assembled structure strongly affected the microphase separation from the crystallization of the PCL segment [49]; (2) The very weak hydrogen bonding interaction of the PLA segment could provide larger pore size of mesoporous carbons with a hydrophobic segment having a similar molecular weight [50]. Therefore, a mesoporous carbon with large pore size could be obtained using this LEL triblock copolymer as a template in this study. Scheme 1 shows that the resol-type phenolic resin acted as the carbon source templated by the LEL triblock copolymer through thermal curing and carbonization procedures. First we discuss the hydrogen bonding and self-assembled nanostructures of phenolic/LEL blends, and then the self-assembled porous structure, surface area, pore volume, and pore size of these very large mesoporous carbons were also investigated in detail. Furthermore, the applications of these large mesoporous carbons in energy storage and CO2 capture were enriched by using this simple ROP approach to synthesize this LEL triblock copolymer as a single template.

## 2. Experimental Section

### 2.1. Materials

The triblock copolymer PLA_440_-*b*-PEO_454_-*b*-PLA_440_ (LEL) was synthesized using dihydroxyl-terminated poly(ethylene oxide) (PEO_454_) as the macroinitiator, D,L-lactic acid (LA) as a monomer, and stannous(II) octoate as a catalyst through ROP. The LA monomer was introduced into the PEO_454_ macroinitiator under N_2_ atmosphere and then stirred at 130 °C for 1 day. The LEL triblock copolymer was dissolved in CH_2_Cl_2_ and then precipitated in n-hexane and dried under vacuum for 2 days [50]. The molecular weight of resol-type phenolic resin was ca. 500 g/mol, which was synthesized though a condensation reaction in NaOH using phenol and formaldehyde [50,54].

### 2.2. The Preparation of Mesoporous Carbon

We prepared various phenolic/LEL mixtures with different ratios dissolved in THF, where the carbon source and the template were resol phenolic resin and LEL triblock copolymer, respectively. The mixtures were stirred for 3 days at room temperature, and then the THF solvent was slowly evaporated at room temperature for the evaporation-induced self-assembly (EISA) approach. The powder was then placed into the oven at 150 °C for 48 h for the thermal curing of phenolic resin by the reaction-induced microporous separation mechanism, and the corresponding mesoporous carbon was obtained using thermal calcination for template removal from room temperature to 700 °C at 1 °C/min, as displayed in Scheme 1.

## 3. Results and Discussion

### 3.1. Characterizations of Phenolic/PLA-b-PEO-b-PLA Blend

**Figure 1:**
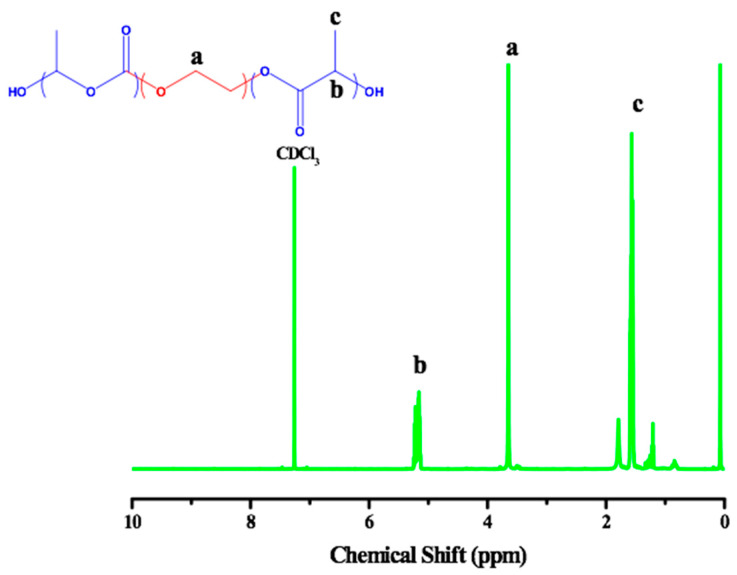
^1^H NMR spectrum of LEL triblock copolymer.

We first used ROP to synthesize the PLA_440_-*b*-PEO_454_-*b*-PLA_440_ triblock copolymer as a template, and its molecular weight was determined using the ^1^H NMR spectrum as displayed in Figure 1. The typical CH_2_ protons (peak a) for the PEO segment were located at 3.65 ppm, and the methine (CH, peak b) and methyl (CH_3_, peak c) protons for the PLA segment appeared at 5.20 and 1.56 ppm, respectively. The molecular weight of this LEL triblock copolymer could be calculated by the peak area ratio of peak a with peak b, and the triblock copolymer polydispersity was 1.15 based on GPC analysis. Scheme 1 displays the preparation of mesoporous carbon templated by LEL triblock copolymer from the phenolic/LEL blends. The corresponding mesophase was gradually formed by the EISA mechanism, further thermal curing, and carbonization for template removal.

**Figure 2:**
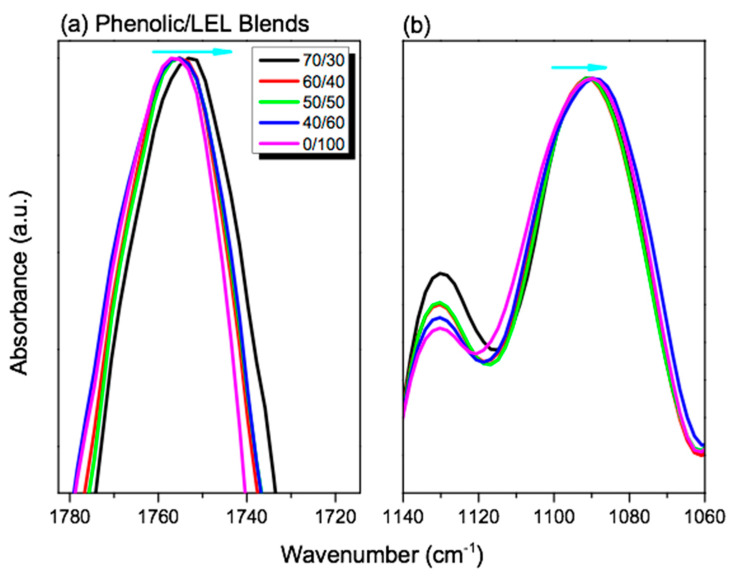
(**a**) C=O and (**b**) C–O–C absorptions of various phenolic/LEL blends based on FTIR spectra recorded at room temperature.

We used FTIR spectra to investigate the hydrogen bonding interaction in phenolic/LEL blends, as shown in Figure 2. The PLA block segment exhibited a free C=O group at 1756 cm^−1^, as shown in Figure 2a, and no shoulder peak corresponding to hydrogen-bonded C=O was observed. However, only the free C=O group was gradually shifted to a lower wavenumber with the increase of phenolic concentration and was located at 1752 cm−1 for the phenolic/LEL = 70/30 blend, indicating a weak hydrogen bonding interaction in the phenolic/PLA binary domain. In our previous studies, we determined that the inter-association equilibrium constant (*K*_A_) for phenolic/PLA blend was smaller than 10, which is quite different to phenolic blends with other carbonyl-based polymers such as PVAc (*K*_A_ = 83) [58], PCL (*K*_A_ = 116) [55], or PAS (*K*_A_ = 64) [59]. Figure 2b displays the C–O–C absorption in phenolic/LEL blends. The PEO segment exhibited C–O–C absorption at 1090 cm^−1^, and this peak shifted to 1088 cm^−1^, which also corresponded to the hydrogen bonding interaction of the phenolic/PEO binary domain. Since ether absorption is highly conformationally sensitive, it was hard to calculate the *K*_A_ value; however, we calculated the *K*_A_ value for phenolic/PEO blend (*K*_A_ = 264) indirectly by using phenolic/PEO/PCL ternary blend [55]. Based on this result, we determined that the OH unit of the phenolic generally preferred to interact with PEO rather than PLA in the phenolic/LEL blends.

**Figure 3:**
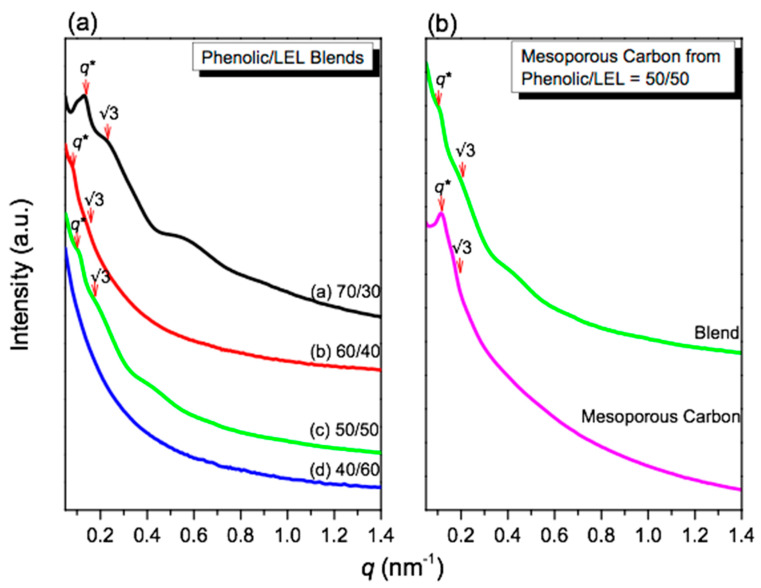
(**a**) SAXS patterns of various phenolic/LEL blends and (**b**) SAXS patterns of mesoporous carbon from phenolic/LEL = 50/50 blend.

Figure 3a presents the SAXS patterns of various phenolic/LEL blends recorded at room temperature. It clearly displays the scattering patterns with the peak ratios of 1:√3 for phenolic/LEL = 70/30, 60/40, and 50/50 blends, corresponding to cylindrical structures. In addition, the first peaks at q* = 0.127 nm^−1^ (*d* = 49.4 nm), 0.074 nm^−1^ (*d* = 84.8 nm), and 0.105 nm^−1^ (*d* = 59.8 nm) for phenolic/LEL = 70/30, 60/40, and 50/50 blends, respectively, suggest that the phenolic/LEL = 60/40 blend possessed the largest *d*-spacing of all these blend systems. When the phenolic/LEL ratio was further decreased to 40/60, it displayed no peak, indicating a disordered structure at this composition.

### 3.2. Analyses of Mesoporous Carbons from Phenolic/LEL Blends

The mesoporous carbon could be obtained by thermal curing at 150 °C for 1 day using phenolic resin and LEL triblock copolymer as the template, which was removed by thermal calcination at 700 °C. Figure 3b compares the SAXS patterns of mesoporous carbon and its corresponding phenolic/LEL = 50/50 blend. We could observe that the peak ratio was maintained at 1:√3, but the first peak was shifted from q^*^ = 0.105 nm^−1^ (*d* = 59.8 nm) to q^*^ = 0.114 nm^−1^ (*d* = 55.1 nm) and became sharp after thermal calcination. This result indicates that the electron density contrast was increased and oxygen or hydrogen was removed to decrease the *d*-spacing by pore formation.

**Figure 4:**
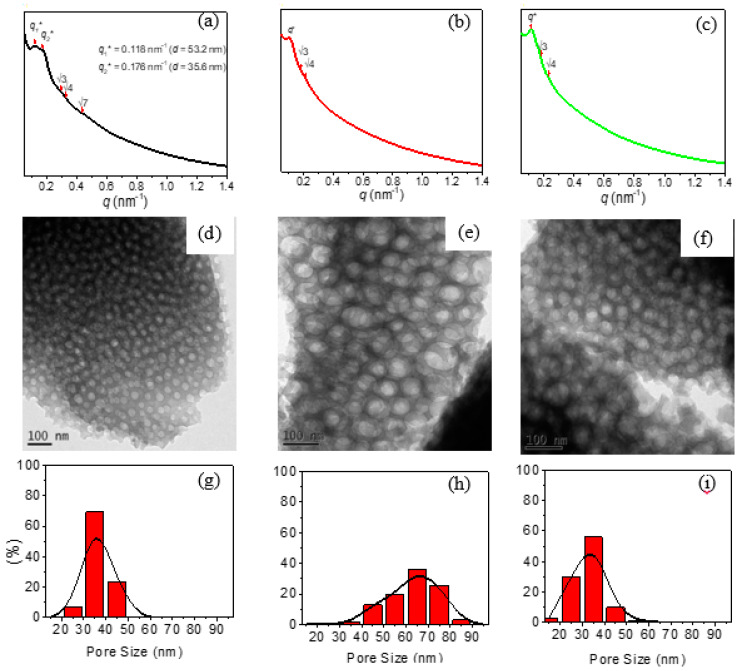
SAXS pattern, TEM image, and the corresponding pore size distribution of mesoporous carbons from phenolic/LED blends of (**a**,**d**,**g**) 70/30, (**b**,**e**,**h**) 60/40, and (**c**,**f**,**i**) 50/50, respectively.

Figure 4 presents the SAXS pattern and TEM images of mesoporous carbons from the corresponding phenolic/LEL blends. Figure 4a displays the SAXS pattern of the mesoporous carbon from phenolic/LEL = 70/30 blend, in which there were two peaks found at low *q* value, suggesting two different pore sizes from the spherical or cylindrical structures. Peak ratios of 1:√3:√4:√7 were observed for the second q* value, corresponding to the cylindrical structure. This was confirmed by the TEM image shown in Figure 4d and the corresponding pore size distribution based on the TEM image exhibited in Figure 4g with 26.5 ± 4.9 nm. The SAXS patterns of the mesoporous carbon from phenolic/LEL= 60/40 and 50/50 blends presented in Figure 4b,c both displayed the peak ratios of 1:√3:√4, indicating the cylindrical structure, as observed by TEM images in Figure 4e,f. The pore size distributions based on TEM images were summarized and are displayed in Figure 4h (54.0 ± 11.6 nm) and Figure 4i (36.8 ± 9.2 nm). The mesoporous carbon with macroporous sizes (>50 nm), indicated the generation of macroporous/mesoporous carbon from the phenolic/LEL= 60/40 blend. Compared with the largest mesoporous carbon from the phenolic/PEO_114_-*b*-PLA_94_ = 60/40 blend with 21.0 nm in our previous work [50], the significantly increased pore size to 54.0 nm from the higher molecular weight of the template from phenolic/PLA_440_-*b*-PEO_454_-*b*-PLA_440_ = 60/40 blend was expected.

**Figure 5:**
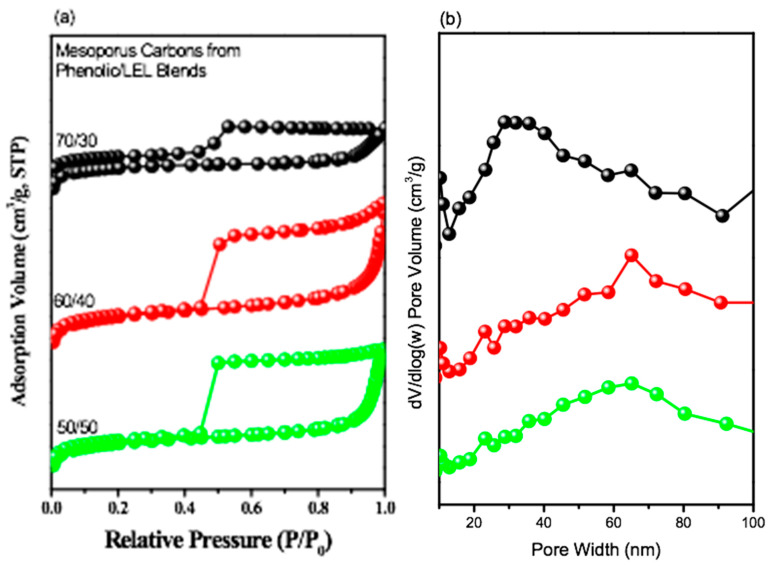
(**a**) N_2_ adsorption isotherms and (**b**) pore size distributions of mesoporous carbons templated by various concentrations of LEL triblock copolymer.

Nitrogen sorption isotherms were used to understand the corresponding pore structures of these mesoporous carbons as displayed in Figure 5a, in which typical IV curves all exhibited H_1_-like hysteresis loops. The relative pressure (*P*/*P*_0_) range from 0.85 to 1.0 was observed for the sharp capillary condensation steps, indicating porous structures with large and cylindrical pores. The results were consistent with the TEM images and SAXS patterns. The corresponding average mesoporous size distribution of these mesoporous carbons based on the Harkins and Jura model were 30.1 ± 11.3, 45.6 ± 11.3, and 44.7 ± 11.4 nm, as displayed in Figure 5b for phenolic/LEL= 70/30, 60/40, and 50/50 blends, respectively. Table 1 summarizes the pore sizes of the mesoporous carbons using BET and TEM analyses, *d*-spacing from SAXS analyses, surface area, and pore volume.

### 3.3. Raman Spectra, CO_2_ Capture Ability, and Electrochemical Analyses of Mesoporous Carbons

**Figure 6:**
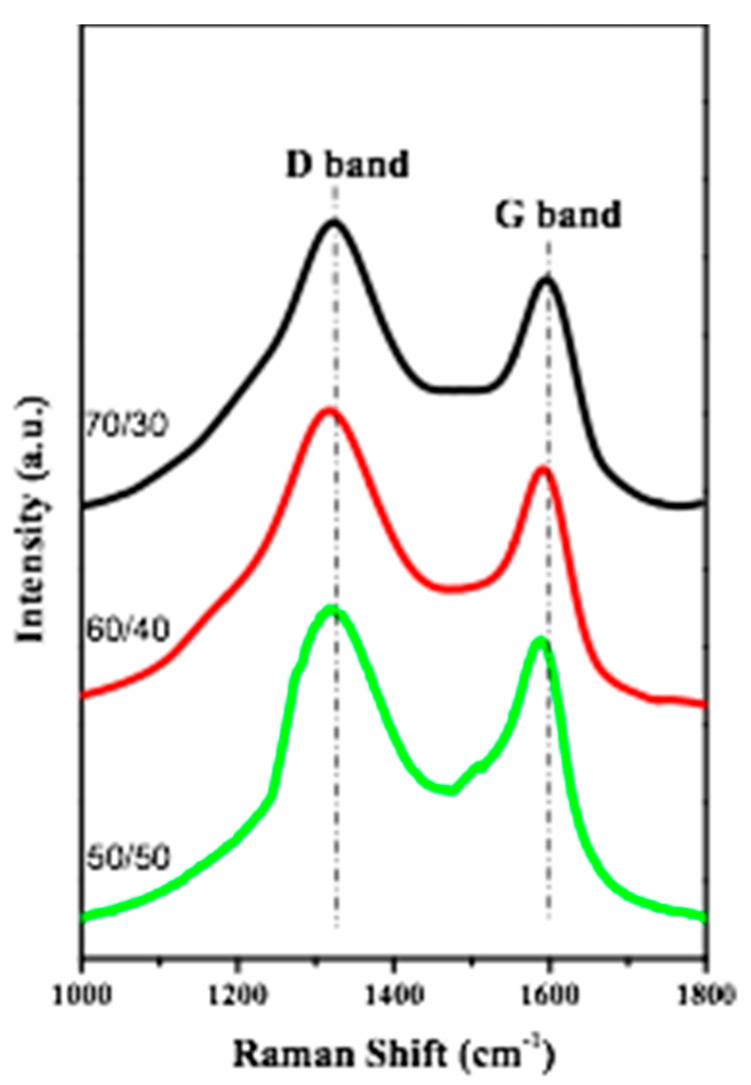
Raman spectra of mesoporous carbons templated by various concentrations of LEL triblock copolymer.

Raman spectra were used to understand the intrinsic behavior of the mesoporous carbons, as displayed in Figure 6. In general, the *I*_D_/*I*_G_ intensity ratios could be used to roughly indicate the degree of graphitization, where the intensity of D and G bands was due to the sp^3^ and sp^2^ hybridized orbital of the C-C bond at 1320 and 1594 cm^−1^, respectively [60,61,62,63]. The *I*_D_/*I*_G_ ratios for the mesoporous carbons from phenolic/LEL = 70/30, 60/40, and 50/50 blends were 2.58, 2.80, and 1.80, respectively, indicating the highly defected structure of mesoporous carbon from phenolic/LEL = 60/40 blend compared with the other mesoporous carbons.

**Figure 7:**
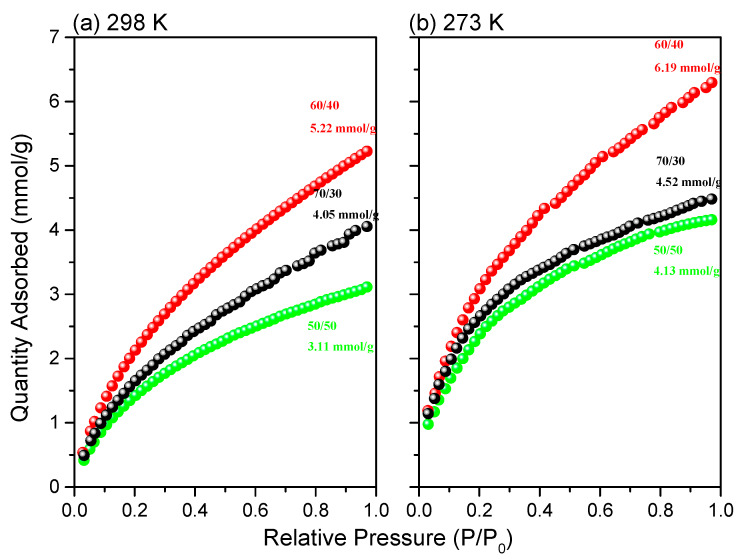
CO_2_ capture behavior properties of mesoporous carbons templated by various concentrations of LEL triblock copolymer at (**a**) 298 K and (**b**) 273 K.

To explore the properties of the high surface area of these mesoporous carbons, we recorded the CO_2_ adsorption isotherms for the mesoporous carbons at 25 and 0 °C, as presented in Figure 7, which exhibited the highest CO_2_ capture ability of 4.05, 5.22, and 3.11 mmol/g at 298 K (Figure 7a) and 4.52, 6.19, and 4.13 mmol/g at 273 K (Figure 7b), respectively. Clearly, the mesoporous carbon from phenolic/LEL = 60/40 blend displayed the highest CO_2_ capture of 5.22 and 6.19 mmol/g at 298 K and 273 K as compared with other mesoporous carbons at the same temperature due to possessing the highest specific surface area and defected structures. This result was also higher than other mesoporous carbons templated by other block copolymers or different materials [6,47,54,61,64,65].

**Figure 8:**
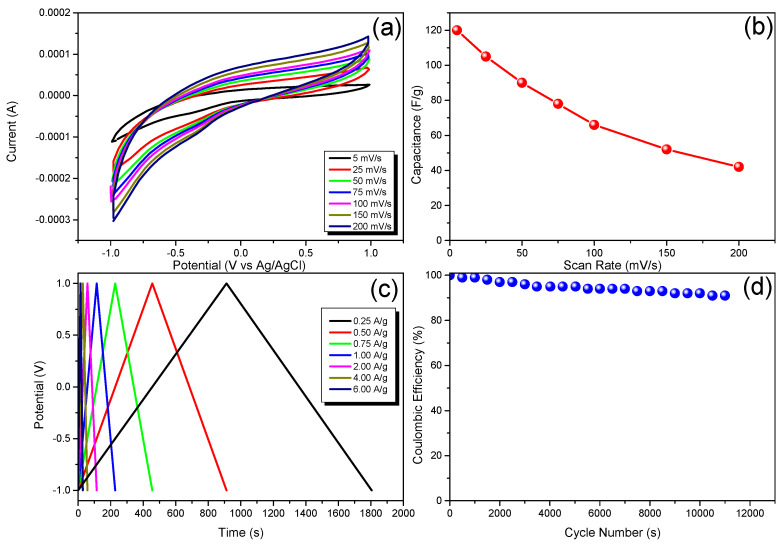
Mesoporous carbon from phenolic/LEL blend (60/40): (**a**) CV curves recorded at various scan rates; (**b**) capacitance plotted versus scan rate; (**c**) charge/discharge curves recorded at various current ranges; (**d**) coulombic efficiency recorded at 20 Ag^−1^ for cycles.

Using these mesoporous carbons in energy storage applications is essential to demonstrate their importance. Therefore, we selected mesoporous carbons from the phenolic/LEL = 60/40 blend to determine their electrochemical performance in a three-electrode cell using 1.0 M KCl as a green medium [66]. Figure 8a displays the CV curves for this mesoporous carbon with a wide potential window range from −1.0 to 1.0 V. The CV curves of the mesoporous carbon were representative of a wide electric double layer capacitor (EDLC) with minor pseudocapacitor (PC) properties. It showed a much higher area of EDLC at all scan rates, as observed in Figure 8b, and the capacitance value at 5 mV·s^−1^ reached 120 F/g. This enhancement of the EDLC performance clearly reflects the major effect of the carbonization technique in providing a higher surface area for electron transfer on the electrode surface. The charge/discharge curves were tested at 2.0 Ag^−1^ at the potential range (−1.0 to 1.0 V), as displayed in Figure 8c. The electrodes displayed efficient stability at 2.0 Ag^−1^ for cycles, with 92% retentions and about 100% coulombic efficiency (Figure 8d). The charge/discharge curves showed typical symmetrical charge/discharge curves at the investigated current densities. Compared to other N-doped carbons and activated carbons, these results offered exceptional negligible IR drop curves with an excellent symmetric triangular shape and the longest potential range [67,68]. Besides, other porous carbons could not show such strong performance. In other words, these results were higher than those of other phenolic carbons used for energy storage, such as lignin-derived porous carbons, which reached a specific capacitance of 100 Fg^−1^ at 5 mV. These results are competitive with the results of other research with similar components [69,70]. The performance of an activated carton box with a surface area over 2700 m^2^ g^−1^ has been investigated, and the obtained capacitance values exhibited a significant decline in retention and cycling abilities [71]. Our results are also much higher compared to other activated bamboo-like carbons composited with metal oxides [72]. Other comparable materials are summarized in Table S1 [73,74,75,76,77,78]. Therefore, we consider this mesoporous carbon to be a promising candidate for energy storage applications.

## 4. Conclusions

Ultra-large mesoporous carbon (>50 nm) with high surface area (>600 m^2^/g) was successfully prepared using resol as the carbon source and templated using a high-molecular-weight LEL triblock copolymer as a single template. The self-assembled cylindrical structure of mesoporous carbons could be mediated by the hydrogen bonding interaction in the phenolic/PEO and phenolic/PLA phases after the thermal curing and carbonization procedures. The larger defected structure of mesoporous carbon from higher *I*_D_/*I*_G_ value based on Raman spectra possessed higher CO_2_ capture ability (6.19 mmol/g at 273 K), efficient capacitance properties (120 F/g at 5 mV s^−1^), and excellent stability of 92% after many cycles. This study provides a facile method for the synthesis of large mesoporous carbon templated by LEL triblock copolymer for electrochemical and CO_2_ capture applications.

## Figures and Tables

**Table 1:** Physical properties of ultra-large mesoporous carbons templated by phenolic/PLA-*b*-PEO-*b*-PLA blends.

Phenolic/LEL	*d* Spacing (X-ray, nm)	Pore Size (BET, nm)	Pore Size (TEM, nm)	*S*_BET_ (m^2^/g)	*V*_Total_ (cm^3^/g)	*V*_Meso_ (cm^3^/g)
70/30	51.2	30.1 ± 11.3	26.5 ± 4.9	441.3	0.27	0.18
60/40	61.6	45.6 ± 11.3	54 ± 11.6	609.7	0.48	0.23
50/50	54.9	44.7 ± 11.4	36.8 ± 9.2	564.2	0.42	0.19

## Supplementary Materials

The following are available online at https://www.mdpi.com/2073-4360/12/5/1193/s1, Table S1: Comparison of different carbons with surface area and capacitance results.
