# Supplementary material for: High-Molecular-Weight PLA-b-PEO-b-PLA Triblock Copolymer Templated Large Mesoporous Carbons for Supercapacitors and CO2 Capture
Source: Polymers (Basel). 2020 May 23;12(5):1193. doi: 10.3390/polym12051193 (PMC7284743; doi:10.3390/polym12051193)
Supplement: Supplementary file 1 [file polymers-12-01193-s001.pdf]

Supporting Information

High Molecular Weight of PLA-*b*-PEO-*b*-PLA Triblock Copolymer Templated Large Mesoporous Carbons for Supercapacitors and CO_2 Capture

Mohamed Gamal Mohamed,[a] Wei-Shih Hung,[a] Ahmed F. M. EL-Mahdy,[a] Mahmoud MM Ahmed,[a] Lizong Dai,[b] Tao Chen,[c] and Shiao-Wei Kuo[a,d*]

[a]Department of Materials and Optoelectronic Science, Center of Crystal Research, National Sun Yat-Sen University, Kaohsiung, 80424, Taiwan. mgamal.eldin12@yahoo.com (M.G.M.) m063100027@student.nsysu.edu.tw (W.S.H.) ahmed1932005@gmail.com (A.F.M.E.M.) kuosw19760501@gmail.com (M.M.M.A.) and kuosw@faculty.nsysu.edu.tw (S.W.K.)

[b]Fujian Provincial Key Laboratory of Fire Retardant Materials, College of Materials, Xiamen University, Xiamen, 361005, China. lzdai@xmu.edu.cn (L.D.)

[c]Ningbo Institute of Material Technology and Engineering, Chinese Academy of Science, Zhongguan West Road 1219, 315201 Ningbo, China. tao.chen@nimte.ac.cn *(T.C.)*

[d]Department of Medicinal and Applied Chemistry, Kaohsiung Medical University, Kaohsiung 807, Taiwan

Correspondence: kuosw@faculty.nsysu.edu.tw (S.-W.K.); Tel.: +886-7-525-4099 (S.-W.K.)

Experimental section

Characterization

FTIR spectra were recorded using a Bruker Tensor 27 FTIR spectrophotometer and the conventional KBr disk method; 32 scans were collected at a spectral resolution of 4 cm^{-1}. The films used in this study were sufficiently thin to obey the Beer-Lambert law. Wide-Angle X-ray diffraction (WAXD) pattern was obscured from the wiggler beamline BL17A1 of the National Synchrotron Radiation Research Center (NSRRC), Taiwan. A triangular bent Si (111) single crystal was used to obtain a monochromated beam having a wavelength (λ) of 1.33 Å. Cross-polarization with MAS (CPMAS) was used to acquire ^{13}C NMR spectral data at 75.5 MHz. The CP contact time was 2 ms; 1H decoupling was applied during data acquisition. The decoupling frequency corresponded to 32 kHz. The MAS sample spinning rate was 10 kHz. Transmission electron microscope (TEM) images were obtained with a JEOL JEM-2010 instrument operated at 200 kV. BET surface area and porosimetry measurements of the prepared samples (ca. 40-100 mg) were performed using a Micromeritics ASAP 2020 Surface Area and Porosity analyzer. Nitrogen isotherms were generated through incremental exposure to ultrahigh-purity N_2 (up to ca. 1 atm) in a liquid nitrogen (77 K) bath. Surface parameters were determined using BET adsorption models in the instrument's software. TGA was performed using a TA Q-50 analyzer under a flow of N_2 atmosphere. The samples were sealed in a Pt cell and heated from 40 to 800 °C at a heating rate of 20 °C min^{-1} under a flow of N_2 atmosphere at a flow rate of 60 mL min^{-1}. The Raman spectra were investigated using Horiba Jobin-Yvon HR800 Raman Spectrometer with 633 nm laser, 10 sec accumulated scans repeated for 20 times and 50x magnification lens. The electrochemical performances were performed using Zahner Zennium E electrochemical workstation with three electrodes configurations using Ag/AgCl as a reference electrode and 1.0 M KCl solution as an aqueous medium. The working electrode was an ITO substrate coated by the slurry of the tested material. The cyclic voltammetry (CV) was tested over the range between 0.0 and 1.0 V and the

scan rates were investigated from 5 mV/s up to 200 mV/s. Similarly, the charge and discharge (CD) cycles were tested at the same potential range with different current densities from 1 to 20 μA/ 0.5 cm^3. The capacitances (C) were calculated using the reported equations for CV and CD respectively.

Table S1: Comparison of different carbons with surface area and capacitance results

Materials	S.S.A m^2g^{-1}	Capacitance	Ref
Heating exfoliated GIC at 2000 °C	50	90 Fg^{-1} @ $5mVs^{-1}$	71
Pyrolysis of pistachio nutshell biomass	1900	45 Fg^{-1} @ 1 mVs^{-1}	72
activated carbon and carbon fiber yarns	1600	45.2 mF cm^{-1}@ 2 mVs^{-1}	73
Mesoporous Nitrogen-Doped Hollow Carbon Nanoplates with Uniform Hexagonal Morphologies	460	95 Fg^{-1} @ 1.0 Ag^{-1}	74
Carbon nanotube composite with activated carbon 15 min	-	60.75 F cm^{-3}	75
Nanoholes on Single-Walled Carbon Nanohorn	1020	8.6 μF cm^{-2}	76
